# Tribbles Pseudokinase 2 (TRIB2) Regulates Expression of Binding Partners in Bovine Granulosa Cells

**DOI:** 10.3390/ijms22041533

**Published:** 2021-02-03

**Authors:** Aly Warma, Jacques G. Lussier, Kalidou Ndiaye

**Affiliations:** Département de Biomédecine Vétérinaire, Centre de Recherche en Reproduction et Fertilité (CRRF), Faculté de Médecine Vétérinaire, Université de Montréal, 3200 Rue Sicotte, St-Hyacinthe, QC J2S 2M2, Canada; aly.warma@umontreal.ca (A.W.); jacques.lussier@umontreal.ca (J.G.L.)

**Keywords:** TRIB2, granulosa cells, yeast two-hybrid, INPPL1, INHBA, RAB14, CALM1, NT5E, SCD, CRISPR/Cas9

## Abstract

Members of the Tribbles (TRIB) family of pseudokinases are critical components of intracellular signal transduction pathways in physiological and pathological processes. TRIBs, including TRIB2, have been previously shown as signaling mediators and scaffolding proteins regulating numerous cellular events such as proliferation, differentiation and cell death through protein stability and activity. However, the signaling network associated with TRIB2 and its binding partners in granulosa cells during ovarian follicular development is not fully defined. We previously reported that TRIB2 is differentially expressed in growing dominant follicles while downregulated in ovulatory follicles following the luteinizing hormone (LH) surge or human chorionic gonadotropin (hCG) injection. In the present study, we used the yeast two-hybrid screening system and in vitro coimmunoprecipitation assays to identify and confirm TRIB2 interactions in granulosa cells (GCs) of dominant ovarian follicles (DFs), which yielded individual candidate binding partners including calmodulin 1 (CALM1), inhibin subunit beta A (INHBA), inositol polyphosphate phosphatase-like 1 (INPPL1), 5′-nucleotidase ecto (NT5E), stearoyl-CoA desaturase (SCD), succinate dehydrogenase complex iron sulfur subunit B (SDHB) and Ras-associated protein 14 (RAB14). Further analyses showed that all TRIB2 binding partners are expressed in GCs of dominant follicles but are differentially regulated throughout the different stages of follicular development. CRISPR/Cas9-driven inhibition along with pQE-driven overexpression of TRIB2 showed that TRIB2 differently regulates expression of binding partners, which reveals the importance of TRIB2 in the control of gene expression linked to various biological processes such as proliferation, differentiation, cell migration, apoptosis, calcium signaling and metabolism. These data provide a larger view of potential TRIB2-regulated signal transduction pathways in GCs and provide strong evidence that TRIB2 may act as a regulator of target genes during ovarian follicular development.

## 1. Introduction

The Tribbles (TRIB) family of serine/threonine pseudokinases include TRIB1, TRIB2 and TRIB3, which represent atypical members of the serine/threonine kinase superfamily [1,2,3,4] and are homologues of the *Drosophila* pseudokinase “Tribbles” [5,6]. Pseudokinase proteins play critical nonenzymatic roles in the regulation of diverse cellular processes and have been associated with numerous key biological pathways [7]. Although they lack canonical phosphotransferase activity, pseudokinases play important roles as signal transduction mediators and protein scaffolds promoting degradation or stability of their target substrates. Instead of direct phosphorylation of target proteins, Tribbles interact with various signaling molecules and transcription factors and act as adaptors in signaling pathways for important cellular functions and neoplastic transformations [8,9]. Functionally, Tribbles pseudokinases share a PEST domain, which mediates protein degradation in the N-terminal region, a Trib domain that is homologous to protein serine/threonine kinases but lacks a catalytic function, a constitutive photomorphogenesis 1 (COP1) site allowing key proteins to be targeted to the proteasome for degradation, and a MAPK (mitogen-activated protein kinase)/ERK (extracellular signal-regulated kinase) Kinase 1 (MEK1) binding site that modulates MAPK activity [7,10]. The MEK1 and COP1 binding sites located at the C-terminal region facilitate, respectively, TRIB-mediated modulation of the MAPK/ERK signal transduction pathway through increased ERK phosphorylation, and proteasomal degradation by interacting with COP1 E3 ubiquitin ligase [7,11,12]. Tribbles family members can therefore modulate the ubiquitin–proteasome system, thereby stabilizing protein substrates as previously shown with TRIB3 [13]. Specifically, TRIB3 inhibits ubiquitylation and degradation of promyelocytic leukemia protein (PML) in acute promyelocytic leukemia progression by repressing proteasome recruitment and interaction with PML and by inhibiting the ubiquitin proteasome system activity [13]. As for TRIB2 in particular, it was also associated with ERK signaling pathway in ovarian granulosa cells as increased ERK1/2 phosphorylation was observed in TRIB2-overexpressed cells [14].

*TRIB2* mRNA was shown to be rapidly induced by mitogens with a short half-live and expressed in a cell-specific manner [1,2,3,4]. In *Drosophila*, Tribbles controls string/CDC25 phosphatase that is required for the progression of G2 cell cycle stage into mitosis. Loss of TRIB2 in *Drosophila* is associated with increased proliferation, whereas overexpression slows the cell cycle and alters fertility [5]. Furthermore, TRIB2 binds to and facilitates the degradation of CCAAT/enhancer-binding protein alpha (C/EBP-α) in a process proposed to be a key feature of TRIB2-induced acute myelogenous leukemia [15]. TRIB2 as well as other TRIB members control hematopoietic cell development and regulate cellular function via protein interactions and regulation of the activity and degradation of target proteins. Various studies have reported proteins interacting with TRIB2, including COP1 [12], C/EBPα [15], C/EBPβ [16], MEK1 [17], MKK7 [2] and AKT1/2 [18]. Moreover, it was shown that TRIB members regulate signal transduction pathways including the Ras-Raf-MEK-ERK MAPK pathway in both normal and malignant cells [19]. These reports indicate that TRIB members are involved in numerous cellular processes, could regulate the expression of target genes in different cell types including reproductive cells and control various cellular processes.

We previously reported the regulation of the expression of Tribbles genes in granulosa cells of ovarian follicles during follicular development [14]. Overall, the expression of genes in the bovine preovulatory follicle assures its growth but abrogates the development of other potentially ovulatory follicles through atresia. During the processes of follicular development and ovulation, steroidogenic cells, including granulosa cells, play a crucial role in the maturation and release of the oocyte with timely production of steroid hormones and growth factors that affect the fate of the ovarian follicle. A proper regulation of granulosa cells activity is therefore crucial in order to sustain follicular and oocyte growth for reproductive success. However, despite the fact that a large number of genes have been identified through the sequencing of the bovine genome, the biological functions and signaling mechanisms of many genes in granulosa cells have still not been fully investigated. In light of this, to identify novel TRIB2 interacting partners in granulosa cells and better understand TRIB2 association with various pathways, we used a yeast two-hybrid (Y2H) system to screen a cDNA library of granulosa cells obtained from dominant follicles. Subsequent manipulations of TRIB2 expression via CRISPR/Cas9 inhibition and pQE overexpression were performed to elucidate the effects of TRIB2 in the regulation of interacting binding partners in granulosa cells identified through the Y2H screening.

## 2. Results

### 2.1. Yeast Two-Hybrid (Y2H) Screening Revealed Potential TRIB2 Partners in Granulosa Cells

To identify TRIB2 binding partners in granulosa cells, a Y2H screening was performed. The Y2HGold yeast strain was transformed either with pGBKT7 empty vector as control or the plasmid construct containing TRIB2 (pGBKT7-TRIB2) and was spread on selective media to verify for toxicity and autoactivation analyses. The pGBKT7-TRIB2 construct was not toxic to the Y2HGold yeast strain and TRIB2 did not, by itself, activate the transcription of reporter and selection genes (*AUR-C*, *ADE2*, *HIS3*, and *MEL1*), since no colonies grew when TRIB2 construct was plated in the presence of aureobasidin A antibiotic. Mating of the Y2HGold[pGBKT7-TRIB2] construct with the Y187[pGADT7-GC-cDNA] library resulted in the presence of zygotes, which indicated potential interactions between the bait (TRIB2) and prey proteins contained in the dominant follicle granulosa cells (GCs)-cDNA library. Potential positive yeast colonies were used to test for the presence or absence of a cDNA insert. The presence of a cDNA insert indicated a true positive, suggesting a potential partner for TRIB2, while the absence of an insert indicated a false positive. Plasmids from positive colonies were purified from yeast colonies containing an insert, amplified by PCR and sequenced. Sequence analyses from the Y2H screening of the dominant follicle GC-cDNA library resulted in the identification of seven TRIB2 binding partners in GCs (Table 1).

### 2.2. TRIB2 Physically Interacts with Its Binding Partners Identified with Y2H Screening

**Figure 1:**
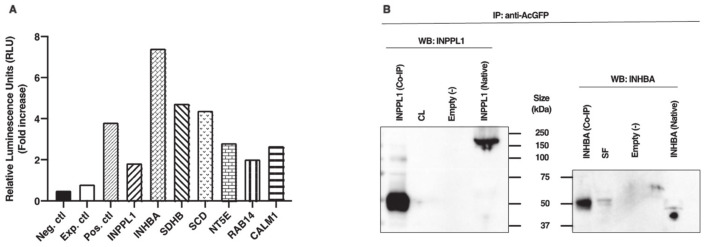
(**A**) Chemiluminescence analyses and confirmation of TRIB2 interaction with its binding partners. Using the ProLabel enzyme complementation assay, luminescent signals (expressed in relative luminescent units (RLUs)) of TRIB2 interactions were compared to a positive interaction (Pos. ctl) consisting of pAcGFP1-53 and ProLabel-T, to an experimental control (Exp. ctl) consisting of pAcGFP1-TRIB2 and ProLabel-empty vector, and a negative control (Neg. ctl). HEK cells were co-transfected with the respective plasmid constructs. Fold induction in ProLabel enzymatic activity was measured after 60 min of the substrate addition compared to the experimental control (fold increase ≥ 2.3 for all partners compared to Exp. ctl). (**B**) Coimmunoprecipitation and Western blot analyses confirmed TRIB2 interactions. Following HEK transfection with TRIB2 bait plasmid and appropriate inositol polyphosphate phosphatase-like 1 (INPPL1) or inhibin subunit beta A (INHBA) prey plasmids, protein extracts were subjected to co-immunoprecipitation (IP) and Western blotting using anti-INPPL1 and anti-INHBA. The results revealed the presence of INPPL1 (left panel) and INHBA (right panel) confirmed physical interaction with TRIB2.

TRIB2 binding partners identified in this study are calmodulin 1 (CALM1), inhibin subunit beta A (INHBA), inositol polyphosphate phosphatase-like 1 (INPPL1, also known as SH2-containing inositol 5′-phosphatase 2 (SHIP2)), 5′-nucleotidase ecto (NT5E), stearoyl-CoA desaturase (SCD), succinate dehydrogenase complex iron sulfur subunit B (SDHB), and RAS-associated protein 14 (RAB14). In order to further confirm Y2H results in a mammalian cell model, physical interactions between TRIB2 and candidate partners were analyzed in vitro using HEK 293 cells followed by coimmunoprecipitation and ProLabel enzyme complementation assay. Using this assay, relative luminescence signals of TRIB2 interaction with its partners were compared to a reference positive interaction, an experimental control and a negative control. Similar to the positive control, all relative luminescent unit (RLU) signals of TRIB2 binding partners were significantly increased as compared to the experimental and negative controls, confirming a physical interaction with TRIB2 (Figure 1A). After 60 min of incubation with the substrate, induction of ProLabel enzymatic activity in HEK cells co-transfected with TRIB2 and each TRIB2 partner increased by at least 2.3-fold as compared to the experimental control (Figure 1A). As reference, induction of ProLabel enzymatic activity increased in the positive control by 4.7-fold, by 9.2-fold with INHBA, which displayed the strongest ProLabel activity as a result of interacting with TRIB2 and by 2.3-fold with INPPL1, which induced the weakest ProLabel enzymatic activity as compared to the experimental control.

Further confirmation analyses were performed using co-immunoprecipitation (IP) followed by Western blotting using antibodies against INPPL1 and INHBA. These two partners were chosen based on the complementation assay results showing the weakest and strongest luminescence data for INPPL1 and INHBA, respectively. Following cotransfection of HEK cells with constructs of TRIB2 and either INPPL1 or INHBA, protein extracts were subjected to co-IP with anti-AcGFP antibodies followed by Western blot analyses with anti-INPPL1 or anti-INHBA. The results confirmed the presence of INPPL1 protein migrating at around 50 kDa in the sample subjected to co-IP while the protein extracts sample not subjected to co-IP showed the full size of INPPL1 migrating at around 160 kDa (Figure 1B, left panel). An additional band at around 100 kDa was also observed in the sample subjected to co-IP along with the 50 kDa band. This result suggests that INPPL1/SHIP2 could undergo cleavage to generate an active 50 kDa form from the 160 kDa form either prior or following interaction with TRIB2. A similar 51 kDa form of INPPL1/SHIP2 was previously reported and proposed to be the phosphorylated form of INPPL1 [20]. As expected, an empty vector used as negative control did not show any presence of INPPL1 protein expression (Figure 1B); however, the corpus luteum (CL) used as positive control did not show any INPPL1 protein presence either, although it was shown to express the strongest amount of INPPL1 mRNA. Likewise, INHBA was observed following Western blotting at around 50 kDa in the sample subjected to co-IP as well as in small follicle sample (SF) (Figure 1B, right panel). In the protein extracts sample not subjected to co-IP, INHBA was present at the expected size of 44 kDa, while the empty vector showed no INHBA protein (Figure 1B).

### 2.3. TRIB2 Partners Are Differentially Regulated during Follicular Development

**Figure 2:**
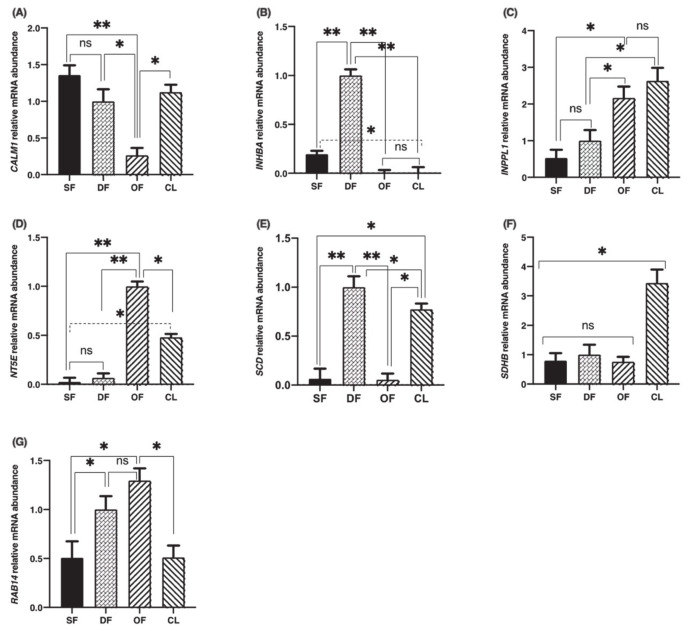
Calmodulin 1 (*CALM1)*, inhibin subunit beta A (*INHBA*), inositol polyphosphate phosphatase-like 1 (*INPPL1*)*,* 5′-nucleotidase ecto *(NT5E)*, stearoyl-CoA desaturase (*SCD)*, succinate dehydrogenase complex iron sulfur subunit B (*SDHB)* and Ras-associated protein 14 *(RAB14)* mRNA expressions in bovine granulosa cells. Total RNA extracts of bovine granulosa cells from small follicles (SF; *n* = 3), dominant follicles (DF) obtained at day 5 of the estrous cycle (*n* = 4), ovulatory follicles (OF) isolated 24 h posthuman chorionic gonadotropin (hCG) (*n* = 4), and corpora lutea (CL) obtained at day 5 of the estrous cycle (*n* = 3) were analyzed by RT-qPCR for *CALM1, INHBA, INPPL1, NT5E, SCD, SDHB, RAB14* and *RPL19* (as reference gene) mRNA expression. *CALM1* (**A**)*, INHBA* (**B***), INPPL1* (**C**)*, NT5E,* (**D**)*, SCD* (**E***), SDHB* (**F***)* and *RAB14* (**G**) relative amounts were normalized with respect to *RPL19* and the results are presented as least-square means ± SEM. *, *p* < 0.05; **, *p* < 0.01.

In order to investigate the regulation of TRIB2 binding partners’ expressions during follicular development, total RNA extracts of bovine granulosa cells from small follicles (SF), day-5 dominant follicles (DF), ovulatory follicles (OF) isolated 24 h posthuman chorionic gonadotropin (hCG) and day-5 corpora lutea (CL) were analyzed by RT-qPCR for *CALM1, INHBA, INPPL1, NT5E, SCD, SDHB* and *RAB14* using specific primers. Corresponding mRNA for all partners was present in dominant follicles with varying abundance. *CALM1* expression was strong in SF, DF and CL while downregulated in OF (Figure 2A; *p* < 0.05). *INHBA* expression was strongest in DF and significantly weaker in SF and even so in OF and CL (Figure 2B; *p* < 0.01). *INHBA* expression was still stronger in SF as compared to OF and CL (Figure 2B; *p* < 0.05). This result confirms previously reported data regarding INHBA regulation during follicular development and its importance in follicular growth [21]. There was no significant difference in *INPLL1* expression between SF and DF while *INPLL1* was significantly stronger in OF and CL as compared to DFs and SFs, displaying a gradual increase from SF through CL (Figure 2C; *p* < 0.05), indicating a possible INPPL1 role in tissue growth and maintenance. There was no significant difference in *INPPL1* expression between OF and CL (Figure 2C). For *NT5E*, steady-state mRNA expression was significantly stronger in OF as compared to SF and DF (Figure 2D; *p* < 0.01) and to CL (Figure 2D; *p* < 0.05), while *NT5E* was stronger in CL as compared to SF and DF (Figure 2D; *p* < 0.05). This result suggests an active involvement of NT5E in the ovulation process or the initiation of granulosa cells differentiation into luteal cells. *SCD* expression was significantly stronger in the DF than in any other stage of follicular development—namely, in SF and OF (Figure 2E; *p* < 0.01) and in the CL (Figure 2E; *p* < 0.05), supporting a role in the growth and maintenance of the dominant follicle. *SCD* downregulation in the OF post-hCG injection was followed by a significant increase in the CL, which was also stronger than in the SF (Figure 2E; *p* < 0.01), suggesting *SCD* importance in the function of CL. As for *SDHB*, its expression was stronger in the CL as compared to any stage of follicular development (Figure 2F; *p* < 0.05), while there was no difference among SF, DF and OF samples, suggesting a major role in corpus luteum function as well. *RAB14* increased significantly in DF and OF from SF and decreased in the CL (Figure 2G; *p* < 0.05), indicating a role in the progression of follicular growth and the onset of ovulation. Overall, because these TRIB2 binding partners have been shown previously to have key functions in ovarian development (as detailed in the Discussion, below), their interactions with TRIB2 that we demonstrate here likely reveal a role in mediating TRIB2 regulation of ovarian development.

### 2.4. Effects of TRIB2 Inhibition and Overexpression on Its Binding Partners

**Figure 3:**
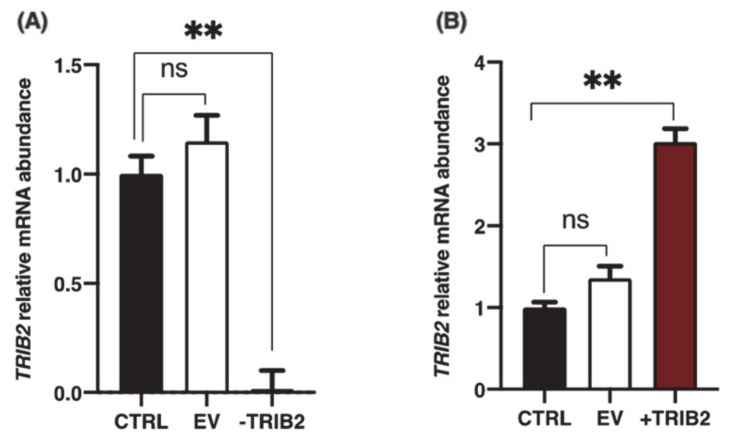
Inhibition and overexpression of TRIB2 in granulosa cells (GCs). (**A**) sgRNA with 96.1% efficiency at directing Cas9-mediated cleavage of *TRIB2* mRNA was identified as described in Materials and Methods and previously reported [14]. TRIB2 knockdown was confirmed by RT-qPCR showing that TRIB2 was significantly reduced in GCs via CRISPR/Cas9 as compared to the control and empty vector. (**B**) The pQE-Trisystem was used to overexpress TRIB2 in GCs as described in Materials and Methods and previously reported [14]. TRIB2 overexpression in GCs was confirmed by RT-qPCR as compared to control and empty vector. **, *p* < 0.01.

**Figure 4:**
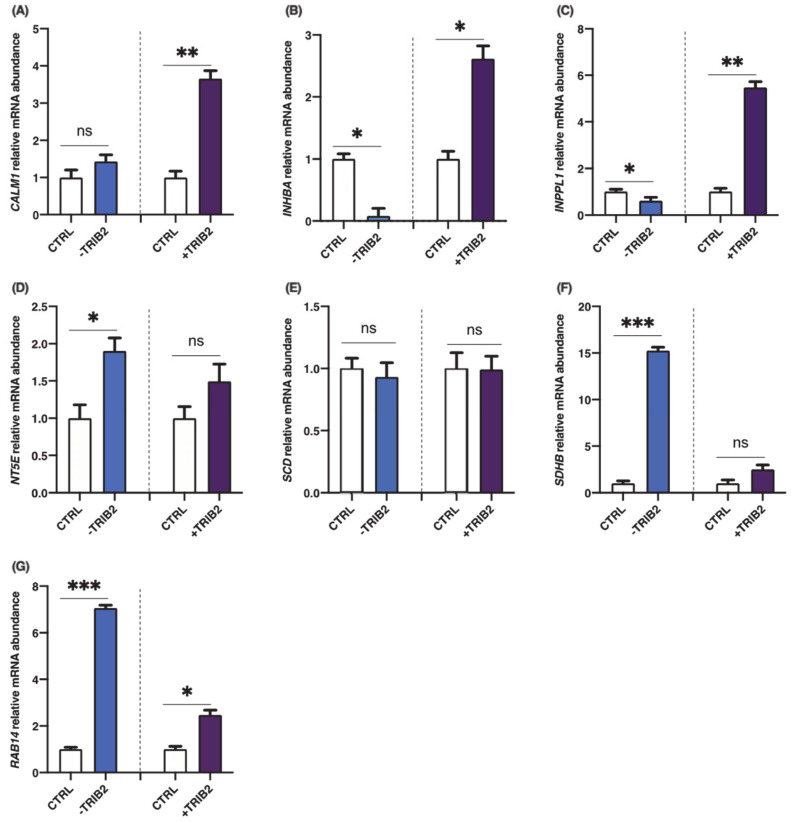
Effects of TRIB2 inhibition and overexpression on *CALM1, INHBA*, *INPPL1*, *NT5E*, *SCD*, *SDHB* and *RAB14*. TRIB2 manipulation (inhibition or overexpression) leads to significant changes in binding partners expression. *INPPL1* and *INHBA* mRNA were significantly decreased in TRIB2-inhibited GCs and significantly increased in TRIB2-overexpressed GCs. *CALM1* was also increased following TRIB2 overexpression but did not change following TRIB2 inhibition, while *NT5E* and *SDHB* were increased following TRIB2 inhibition but did not change following TRIB2 overexpression. *RAB14* was increased in TRIB2 inhibition and overexpression while there was no effect on *SCD* following TRIB2 manipulation. Relative amounts for *CALM1* (**A**)*, INHBA* (**B***), INPPL1* (**C**)*, NT5E,* (**D**)*, SCD* (**E***), SDHB* (**F***)* and *RAB14* (**G**) were normalized with respect to *RPL19* and the results are presented as least-square means ± SEM. *, *p* < 0,05; **, *p* < 0.01; ***, *p* < 0.001).

To investigate the effects of TRIB2 inhibition and overexpression on its binding partners, total RNA was extracted from cultured granulosa cells at day 6 following CRISPR/Cas9-induced inhibition or pQE-induced overexpression of TRIB2. Inhibition and overexpression of TRIB2 were confirmed by RT-qPCR. The results confirmed TRIB2 downregulation via CRISPR/Cas9 (Figure 3A; *p* < 0.01), while TRIB2 was overexpressed using the pQE system (Figure 3B; *p* < 0.01). These data confirming TRIB2 inhibition and overexpression in our model were previously published [14]. Subsequent analyses demonstrated that TRIB2 inhibition did not change *CALM1* abundance while TRIB2 overexpression resulted in significant increase in *CALM1* abundance (Figure 4A; *p* < 0.01). For *INHBA* (Figure 4B) and *INPPL1* (Figure 4C), TRIB2 inhibition led to similar downregulation of their expression (*p* < 0.05). In contrast, TRIB2 overexpression resulted in increased expression of both *INHBA* (Figure 4B; *p* < 0.05) and *INPPL1* (Figure 4C; *p* < 0.01) suggesting a strong TRIB2 modulatory role on these binding partners. TRIB2 inhibition had a positive effect on *NT5E* (Figure 4D; *p* < 0.05) and *SDHB* (Figure 4F; *p* < 0.001) expression; however, there was no significant effect of TRIB2 overexpression in *NT5E* and *SDHB* relative abundance. As for *SCD* (Figure 4E), there was no effect either way following TRIB2 inhibition and overexpression. Finally, *RAB14* displayed a surprising result as both inhibition and overexpression of TRIB2 resulted in increased expression of RAB14 (Figure 4G).

## 3. Discussion

Tribbles pseudokinases act as scaffold proteins, which can bind their substrates and localize them to or from their functions [22]. Moreover, Tribbles gene expression was shown to be tissue- and cell-specific [3]. We previously reported the regulation of Tribbles genes expression in GCs of ovarian follicles during follicular development [23]. Further, our recent published data provided evidence that TRIB2 is a regulator of GC proliferation and that it could affect steroidogenesis and MAPK signaling pathways in these reproductive cells [14]. The current study describes, for the first time to our knowledge, TRIB2 protein interactions in granulosa cells using yeast two-hybrid screening and the effects of TRIB2 on binding partners regulation in the reproductive system using granulosa cells from ovarian follicles. These observations indicate that TRIB2 might be associated with controlling the activity of target genes involved in the growth and/or maintenance of the dominant follicle immediately prior to the ovulatory stage and ovulation. Moreover, the interactions between TRIB2 and binding partners could lead to various events including degradation or activation of target proteins resulting in gene expression changes and possibly contributing to follicular growth, differentiation of steroidogenic cells into luteal cells after ovulation and function of the corpus luteum.

We identified seven individual partners for TRIB2 in bovine granulosa cells and confirmed the interactions by in vitro coimmunoprecipitation followed by ProLabel enzymatic complementation assay and Western blotting. Among TRIB2 partners in GCs, INHBA is known to be expressed in dominant follicles and participates actively in the establishment of the dominant or preovulatory follicles. We showed that INHBA interacts with TRIB2 in granulosa cells and its expression was significantly decreased in TRIB2-inhibited GCs while significantly increased in TRIB2-overexpressed GCs. Previous studies showed greatest expression for *INHBA* in estrogen active follicles [24,25] and an increase in activin-A protein secreted in follicular fluid [26]. Activin promotes granulosa cells proliferation and steroidogenesis and potentiates follicle-stimulating hormone (FSH) actions on granulosa cells by increasing FSH receptor expression [27,28], which underscores a key role for activin-A in the dominant follicle’s development. Our data confirmed expression of *INHBA* mRNA in dominant follicles, in agreement with already published data. The interaction between TRIB2 and INHBA could participates in the activity of granulosa cells and in the growth of the dominant follicles into the ovulatory stage, prior to the luteinizing hormone (LH) surge.

Another TRIB2 binding partner, Inositol polyphosphate phosphatase-like 1 (INPPL1), also known as SH2-containing inositol 5′-phosphatase 2 (SHIP2), is a negative regulator of insulin signaling since its inactivation in mice resulted in increased insulin sensitivity [29] in addition of mediating obesity resistance [30]. INPPL1/SHIP2 expression has been shown in cells of hematopoiesis origins as well as in spermatids of adult mice testis, suggesting a role in maintenance of hematopoietic lineage and spermatogenesis [31]. In our study, *INPPL1* was expressed in granulosa cells of dominant follicles but was stronger in ovulatory follicles and even stronger in the corpus luteum, suggesting that INPPL1 could participate in the transition from dominant to preovulatory follicle and through the ovulation process to luteinization. These observations regarding *INPPL1* expression and regulation throughout follicular growth and corpus luteum formation could also support a role for INPPL1 in tissue maintenance and development. INPPL1 expression was previously shown to be enhanced in cultured dog thyroid cells by thyroid-stimulating hormone (TSH) and epidermal growth factor (EGF) [32]. Similarly, TRIB2 was shown to be induced by TSH as well in dog thyroid cells [1,33] suggesting a link between INPPL1 and TRIB2. We showed here that INPPL1 interacts with TRIB2 in granulosa cells and its expression was significantly decreased in TRIB2-inhibited GCs and significantly increased in TRIB2-overexpressed GCs.

INPPL1 has been suggested to act downstream of phosphoinositide-3′ kinase (PI3K), which is a significant component of intracellular signaling mechanisms [34]. PI3K phosphorylates phosphatidylinositol 4,5-bisphosphate to generate phosphatidylinositol 3,4,5-triphospate (PIP3), which then activates several cellular enzymes, including protein kinase Akt, to regulate cell growth and survival [35]. INPPL1 is able to dephosphorylate the 5-position of PIP3 generated by the PI3-kinase, producing a new second messenger PI-3,4-bisphosphate (PI-3,4-P2) [36]. Irregular expression of INPPL1 may then disrupt the normal PI3K/Akt pathway, which has an important function in controlling cell proliferation and cell death as well as tumor development and progression [37]. Other studies have demonstrated a positive role of INPPL1 in EGF-induced Akt activation and cell migration in breast cancer cells [38]. Earlier studies indicated a positive association between INPPL1/SHIP2 expression and cell proliferation where EGF increases *INPPL1* mRNA expression in thyrocytes [32]. In addition, INPPL1 protein expression correlates with the EGFR expression in proliferating neurospheres involved in the EGF signaling pathway [39]. Overall, these observations combined with our findings support a role of INPPL1 as an effector downstream of TRIB2 in granulosa cells and that the interaction between TRIB2 and INPPL1 could play an important role in granulosa cells proliferation and participate in the growth of the dominant follicle into the ovulatory stage.

Calcium signaling activator calmodulin 1 (CALM1), another partner of TRIB2 in granulosa cells, is a ubiquitous Ca^2+^ receptor protein mediating a large number of signaling processes such as nerve growth, proliferation, inflammation, apoptosis, muscle contraction and intracellular movement in eukaryotic cells [40,41,42,43]. Upon binding of four Ca^2+^ ions, calmodulin undergoes conformational changes, allowing this complex to bind to and activate many enzymes including protein kinases, protein phosphatases, ion channels, Ca^2+^ pumps, nitric oxide synthase, inositol triphosphate kinase and cyclic nucleotide phosphodiesterase [44,45]. Since calmodulin binds Ca^2+^ in a cooperative fashion, small changes in cytosolic Ca^2+^ levels lead to large changes in the level of active calmodulin and its target proteins [46]. Of interest, CALM1 was shown to negatively regulate Ras activation and is therefore essential in the downregulation of the Ras/Raf/MEK/ERK pathway following its activation [45]. Further, inhibition of calmodulin was shown to participate in inducing ERK activation [47]. The interaction shown here between CALM1 and TRIB2 could play a key role in modulating CALM1 effect in the Ras/ERK signaling pathway, which can affect proliferation of granulosa cells of the dominant follicle.

CALM1 expression was shown to be associated with ovarian cell survival while negatively affecting caspase-3 activation and apoptosis in patients with PCOS [48]. We have shown in this study that CALM1 expression is strong in granulosa cells of small follicles and in the growing dominant follicle, while it was downregulated by the luteinizing hormone in ovulatory follicles prior to ovulation. This result is consistent with previous studies and suggests a role of CALM1 in the proliferation of granulosa cells and follicular growth. We also showed that *CALM1* expression was significantly increased in TRIB2-overexpressed GCs, supporting the hypothesis of CALM1 playing an important role in the activity of granulosa cells and in the growth of the dominant follicle in the ovulatory stage. Our data showed that *CALM1* was also abundantly expressed in the corpus luteum, similar to the stages of small and dominant follicles, suggesting a role in the differentiation of steroidogenic cells into luteal cells and in luteal function. This is supported by the fact that CALM1 was associated with the differentiation of osteoblasts and its downregulation resulted in the suppression of human osteoclasts differentiation [49,50]. Osteoclast differentiation was also induced through the activation of Ca^2+^/calmodulin-dependent protein kinases (CaMKs) following receptor activation by NF-kappaB ligand [51]. However, the exact mechanism and effect of CALM1 in granulosa cells following interaction with TRIB2 remains to be further investigated.

Ecto-5′-nucleotidase (NT5E) is a surface enzyme that is expressed on multiple cells [52]. It is primarily responsible for the dephosphorylation of extracellular adenosine monophosphate (AMP) released by distressed cells into adenosine, an important activator of Ras/Raf/ERK/MAPK and PI3K/Akt pathways [53]. Adenosine plays a major role in cell growth and proliferation [54], and apoptosis [55,56]. Our data demonstrated the expression of NT5E as significantly induced in the ovulatory follicles post-hCG, while its expression was minimal in early stages and preovulatory follicles, suggesting that NT5E might be induced by the LH surge and is likely involved in the ovulation process. NT5E was also observed in dominant follicles, although in much weaker amounts as compared to the ovulatory follicles and the corpus luteum, supporting the idea of a more significant role in the ovulation process, differentiation and luteal formation than in follicular growth. Since TRIB2 inhibition in cultured granulosa cells resulted in a significant increase in *NT5E*, it might indicate that TRIB2 negatively modulates NT5E, which would be consistent with a weak expression of *NT5E* in dominant follicles where TRIB2 is strongly expressed, in contrast with a strong expression of *NT5E* in ovulatory follicles where *TRIB2* expression was shown to be weak [14]. Further analyses would elucidate the mechanisms and signaling cascade affected by TRIB2 interaction with NT5E.

Stearoyl-coenzyme A desaturase (SCD), also named delta 9 desaturase, is a key lipogenic enzyme found in the endoplasmic reticulum and a rate-limiting enzyme in the biosynthesis of monounsaturated fatty acids from saturated fatty acids [57,58]. Saturated free fatty acids have a dose-dependent negative impact on oocyte developmental competence, while monounsaturated free fatty acids appear less harmful [59]. It was demonstrated that SCD inhibition in the presence of stearic acid significantly reduced the developmental competence of oocytes and increased the incidence of apoptosis in bovine cumulus cells [59]. It was also reported that cumulus cells can desaturate the potentially toxic stearic acid into oleic acid via SCD activity, providing a mechanistic insight into how the cumulus cells protect the oocyte against toxicity by saturated fatty acids [59]. Furthermore, it is known that the MAPK3/MAP1 and PIK3R1/Akt pathways are involved, respectively, in the IGF1- and FSH-induced SCD2 expression in rat ovary and that SCD2 can be involved in the regulation of follicular growth and/or oocyte maturation [60]. These reports are very much consistent with our findings that SCD is strongly expressed in the growing dominant follicle, which corroborates observations in our previous study [23] and are consistent with the status of the dominant follicle characterized by modifications necessary for oocyte growth and maturation. In contrast, SCD expression was abrogated in ovulatory follicles and upregulated in the corpus luteum, providing strong evidence of SCD involvement in follicular growth and corpus luteum formation and function. However, TRIB2 manipulations in cultured granulosa cells did not have any effects on SCD expression, suggesting that additional analyses are required to better define the pathways and biological processes affected by TRIB2 interaction with SCD in granulosa cells.

Finally, Ras-associated protein 14 (RAB14) was also identified as a binding partner of TRIB2 in the Y2H screening. RAB14 is a member of the RAS oncogene superfamily of small G-proteins involved in intracellular membrane trafficking and signal transduction [61]. RAB proteins are inactive when bound to GDP but turn to an active form when bound to GTP. Activated RAB proteins recruit effector proteins to the vesicle membrane and promote membrane trafficking [61]. The ability to switch between the GDP-bound inactive state and the GTP-bound active state allows the RAB proteins to function as cargo carriers to recruit or release molecules onto membranes in a specific compartment. More specifically, RAB14 is involved in membrane trafficking between the Golgi complex and endosomes and regulates apical targeting in polarized epithelial cells [62] as it interacts specifically with some apical targeting proteins [63]. Moreover, the family of RAB proteins plays numerous roles during development and progression of cancers [64,65,66,67]. RAB14, in particular, was associated with aggressiveness of ovarian cancer cells since it promoted cell proliferation through the Wnt signaling pathway [68].

It was shown that RAB14 interacts with cellular communication network factor 2/CCN family 2 (CCN2)/connective tissue growth factor (CTGF) through its IGFBP-like domain, indicating a regulation of intracellular membrane trafficking [69] since CCN2 in involved in many cellular functions including proliferation and differentiation of various types of cells [70,71,72]. RAB14 overexpression was also associated with increased growth rate, cell invasion and cell cycle progression, while also increasing expression of cyclins involved in cell cycle progression such as cyclins D and E and CTGF in cell lung cancers [73]. It was proposed that RAB14 increased proliferation and invasion through the Yes-associated protein (YAP) pathway [73]. Similarly, TRIB2 was also shown to affect CTGF since TRIB2 knockdown in HepG2 cells inhibited CTGF, which is known as a target gene of YAP [74,75]. In line with these findings, we previously demonstrated increased CTGF expression in TRIB2-overexpressed granulosa cells, while TRIB2 inhibition resulted in CTGF downregulation [14], suggesting that TRIB2 could regulate different cellular processes through target effectors and binding partners, such as RAB14, and affecting downstream signaling pathways including the YAP pathway.

Furthermore, our data showed expression of RAB14 in granulosa cells of ovarian follicles throughout follicular development, with the strongest expression observed in the stages of dominant and ovulatory follicles, which suggests that RAB14 might be associated with the growth and maintenance of the dominant follicle and involved in the transition to the ovulatory stages. Surprisingly, both TRIB2 inhibition and overexpression in granulosa cells resulted in significant increases in *RAB14* expression, which could be related to the RAB family’s ability to switch between an inactive and active states and to their numerous roles in developing tissues. It is also conceivable that TRIB2 levels relative to the levels of this key partner may dictate tissue-specific effects as was shown for TRIB3, another Tribbles member [2]. Nonetheless, we provide evidence here that RAB14 interacts with TRIB2 and may play an important role in follicular development and ovulation in a pathway likely involving the YAP pathway, as previously suggested [73].

## 4. Materials and Methods

### 4.1. Experimental Animal Model and In Vivo Sample Preparations

The regulation of *TRIB2* expression as well as mRNA of binding partners was studied during follicular development using samples from an in vivo model previously characterized [23]. Following estrous synchronization with PGF_2α_, normal cycling cows were randomly assigned to a dominant follicle group (DF, *n* = 4), or an ovulatory hCG-induced follicle group (OF, *n* = 4). In the DF group, the ovary bearing the DF on the morning of day 5 of the estrous cycle (day 0 = day of estrus) was obtained by ovariectomy. The DF was defined as ≥8 mm in diameter and growing, while subordinate follicles were either static or regressing. The OFs were obtained following an injection of 25 mg of PGF_2α_ on day 7 to induce luteolysis, thereby promoting the development of the DF of the first follicular wave into a preovulatory follicle. An ovulatory dose of hCG (3000 IU, iv; APL, Ayerst Lab, Montreal, QC, Canada) was injected 36 h after the induction of luteolysis, and ovaries bearing the hCG-induced OFs were collected by ovariectomy 24 h post-hCG. Immediately following ovariectomy, follicles were dissected into separate isolates of granulosa cells [23] and stored at −70 °C. Additionally, granulosa cells (GCs) were collected from 2 to 4 mm small follicles (SF), obtained from slaughterhouse ovaries, and a total of three pools of twenty SF each were prepared (SF, *n* = 3). Corpora lutea (CL, *n* = 3) were obtained at day 5 of the estrous cycle and dissected from the ovarian stroma, frozen in liquid nitrogen, and stored at −70 °C. The experimental protocol was reviewed and approved by the Animal Ethics Committee of the Faculty of Veterinary Medicine of the University of Montreal on 16 July 2018 (project identification code: 18-Rech-1959). Cows were cared for in accordance with the Canadian Council on Animal Care guidelines [76].

### 4.2. In Vitro Samples Preparation

#### Inhibition and Overexpression Experiments

The Clustered Regularly Interspaced Short Palindromic Repeats (CRISPR)/CRISPR-associated endonuclease (Cas9) technology was used through the guide-it CRISPR/Cas9 system (Takara Bio) for the cloning and expression of target single guide RNAs (sgRNAs) for *TRIB2* inhibition in GCs as previously reported [14]. A sgRNA with 96.1% efficiency at directing Cas9-mediated cleavage of *TRIB2* mRNA was identified and used for cloning into the pGuide-it-ZsGreen1 vector for plasmid construct and transfection of GCs using the Xfect transfection kit (Takara Bio, Mountain View, CA, USA) as previously reported [14,77]. GCs were collected from slaughterhouse ovaries and cultured in 24-well plates at a density of 5 × 10^4^ cells in DMEM/F12 supplemented with L-glutamine (2 mM), sodium bicarbonate (0.084%), bovine serum albumin (BSA; 0.1%), HEPES (20 mM), sodium selenite (4 ng/mL), transferrin (5 µg/mL), insulin (10 ng/mL), nonessential amino acids (1 mM), androstenedione (100 nM), penicillin (100 IU) and streptomycin (0.1 mg/mL) (*n* = 3 independent experiments with duplicate wells for each treatment). Culture media and supplements were purchased from Wisent (Wisent Bioproducts, St-Bruno, QC, Canada). Nanoparticle complexes from the Xfect transfection kit were applied to GCs and incubated for 9 h at 37 °C and then removed and replaced with complete growth medium. Transfected GCs along with control GCs (transfection with empty vector or no transfection) remained in culture for six days with media replacement every two days. Cells were collected for total RNA extraction for RT-qPCR analyses. Additionally, TRIB2 was overexpressed in GCs as previously reported using the pQE-TriSystem His-Strep2 vector (Qiagen, Toronto, Ontario, Canada) [14]. *TRIB2* inhibition and overexpression were confirmed by RT-qPCR. The effects of CRISPR/Cas9-induced TRIB2 inhibition pQE-driven TRIB2 overexpression were assessed by analyzing the expression of mRNA of corresponding binding partners: *CALM1*, *INHBA*, *INPPL1*, *NT5E*, *SCD*, *SDHB* and *RAB14*.

### 4.3. Yeast Two-Hybrid Assay

#### 4.3.1. Material and Media Legend

The materials and media used for the yeast two-hybrid assay have been previously reported [21,77].

#### 4.3.2. TRIB2 Constructs for Bait Preparation

A *TRIB2* construct was generated by polymerase chain reaction (PCR) amplification of bovine *TRIB2* (NM_178317.3) using primers that amplify the entire PEST domain in the N-terminal region and a portion of the Trib domain, which is homologous to protein serine/threonine kinases but lacks a catalytic function. The PCR products were purified and cloned in frame to the GAL4-DNA binding domain into the pGBKT7 vector to produce a bait plasmid using the Matchmaker Gold Yeast Two-Hybrid System (Takara Bio, Mountain View, CA, USA) as previously described [21]. The bait plasmid (pGBKT7-TRIB2) was used to transform Y2HGold yeast strains and was referred to as Y2HGold[pGBKT7-TRIB2]. Appropriate controls to verify toxicity and autoactivation of constructs were conducted as previously reported [21,77].

#### 4.3.3. Generation of GC-cDNA Library and Construction of the Two-Hybrid Prey Library

A bovine granulosa cells cDNA prey library (GC-cDNA) from dominant follicles was prepared in Y187 yeast strain using the pGADT7-Rec vector. cDNAs were expressed as fusion to the GAL4 activating domain using the Matchmaker library construction and screening kit (Takara Bio User manual PT4085-1) as previously described [21]. Total RNA was isolated from GCs of dominant follicles and used to generate cDNA with Oligo dT (CDSIII) primers. Competent Y187 yeast cells were prepared and co-transformed with pGADT7-Rec plasmid and cDNAs from GCs of dominant follicles. The transformed yeast cells, referred to as Y187(pGADT7-GC) library, were plated, collected after a 5-day incubation at 30 °C and stored as previously reported [21].

#### 4.3.4. Two-Hybrid Library Screening Using Yeast Mating

The screening procedure was performed as previously reported by our laboratory [21]. Briefly, Y2HGold yeast cells carrying the baits plasmids (Y2HGold(pGBKT7-TRIB2)) were mated with Y187 yeast harboring the bovine GC-cDNA library (Y187(pGADT7-GC)). Target prey plasmids responsible for the activation of reporter genes were rescued, isolated and characterized by sequencing. Nucleic acid sequences were verified for the presence of an open reading frame fused in frame to the *GAL4* AD sequence and were compared to those in GenBank to identify binding partners.

#### 4.3.5. Co-IP Confirmation of Protein Interactions

Physical interactions between TRIB2 and candidate partners were confirmed by in vitro coimmunoprecipitation assay using the Matchmaker Co-IP system (Takara Bio, Mountain View, CA, USA). Plasmid constructs containing TRIB2 and potential prey partners were used to co-transfect HEK 293 cells using the CalPhos Mammalian transfection kit (Takara Bio, Mountain View, CA, USA) as recommended by the manufacturer. The potential prey partners tested were calmodulin 1 (CALM1), inhibin subunit beta A (INHBA), inositol polyphosphate phosphatase-like 1 (INPPL1), 5′-nucleotidase ecto (NT5E), stearoyl-CoA desaturase (SCD), succinate dehydrogenase complex iron sulfur subunit B (SDHB) and member RAS oncogene family (RAB14). Cell lysates were prepared and physical interactions between TRIB2 and prey proteins were validated and quantified using the ProLabel enzyme complementation assay through the ProLabel detection kit II (Takara Bio, cat. # 631629) as per the manufacturer’s guidelines. Briefly, lysis/complementation buffer was added to each well to be assayed and a volume of the lysate was transferred to a 96-well plate followed by the addition of the substrate. After incubation for 1 h, luminescent signals were recorded every 5 min for 1 h using a SpectraMax i3 Multi-Mode microplate reader (Molecular Devices, San Jose, CA, USA). Relative luminescence units were plotted as a function of time in order to quantify the relative importance of protein interactions.

#### 4.3.6. Regulation of TRIB2 Partners during Follicular Development

Expression and regulation of TRIB2 partners during follicular development and following hCG injection was analyzed by RT-qPCR using total RNA extracted from bovine GCs collected from follicles at different developmental stages (SF, DF, OF) and CL. Relative mRNA expressions of *CALM1,* *INHBA, INPPL1, NT5E, SCD, SDHB* and *RAB14* were quantified using specific primers (Table 2), and the results were analyzed using the 2^−ΔΔCt^ method [78].

#### 4.3.7. TRIB2 Effects on Expression of Binding Partners

Total RNA from in vitro samples was extracted from GCs at day 6 of culture following CRISPR/Cas9-induced inhibition or pQE1-induced overexpression of TRIB2. Specific *TRIB2* and TRIB2 binding partner PCR primers were used as presented in Table 2. The relative mRNA expressions of *TRIB2* and other targets were calculated using the 2^−ΔΔCt^ method [78] with *RPL19* as reference gene.

### 4.4. Statistical Analysis

Data are presented as mean ± SEM from three or more independent experiments. Different samples or treatments were compared using one-way analysis of variance (ANOVA). When ANOVA indicated a significant difference (*p* < 0.05), the Tukey–Kramer test was used for multiple comparisons of individual means among SFs, DFs, OFs and CL, and for in vitro experiments. Statistical analyses were performed using Prism software v9 for macOS (GraphPad). RT-qPCR data are presented as normalized amounts of respective genes relative to 2^−^^ΔΔCt^.

## 5. Conclusions

We have identified TRIB2 interactions in granulosa cells of dominant follicles and demonstrated the effects of TRIB2 on its binding partners through manipulations of TRIB2 expression in cultured granulosa cells. Overall, the data reported here provide evidence that TRIB2 could regulate the expression of target genes and signaling pathways linked to cell proliferation and growth. Although the mechanisms by which TRIB2 regulate expression of binding partners was not elucidated in this study, the current findings serve as the basis for future studies targeting granulosa cells regulation during the preovulatory stage by using TRIB2-inhibited and -overexpressed granulosa cells to identify pathways affected by TRIB2 and decipher their relevance in follicular development and activity of granulosa cells.

## Figures and Tables

**Table 1:** List of potential binding partners of Tribbles 2 (TRIB2). Plasmids were purified from true positive yeast colonies, amplified by PCR and sequenced. Sequences were analyzed for identity and resulted in 7 different proteins potentially interacting with TRIB2.

Partners	Accession #	Freq.	Ident. (%)	E-value	UniProt ID	Description
CALM1	NM_001242572.1	1	99	0.0	P62157	B.T. Calmodulin 1
INHBA **	NM_174363.2	6	99	0.0	P07995	B.T. Inhibin subunit beta A
INPPL1 **	NM_001191176.2	1	99	0.0	E1BBJ7	B.T. Inositol polyphosphate phosphatase-like 1
NT5E	NM_174129.4	1	92	0.0	Q05927	B.T. 5’-nucleotidase ecto
SCD	NM_173959.4	1	99	0.0	Q9TT94	B. T. Stearoyl-CoA desaturase
SDHB	NM_001040483.1	1	99	0.0	Q3T189	B.T. Succinate dehydrogenase complex iron sulfur subunit B
RAB14	NM_001130754.1	1	99	0.0	Q3ZBG1	B.T. RAB14, member RAS oncogene family

**#** Accession number of the best match found following nucleotide sequence comparison via BLAST search in GenBank. Freq., frequency of cDNA clone identification from yeast two-hybrid prey library; Ident. (%), identity: represents homology estimates of bovine prey cDNA fragments with nucleotide sequences in GenBank database; B.T., *Bos taurus*. ** Partners whose physical interactions with TRIB2 were further confirmed by coimmunoprecipitation and Western blotting.

**Table 2:** Primers used in the expression analyses of *Bos taurus* genes by RT-qPCR.

Gene Names	Primer Sequences (5′–3′) *	Accession #	AS (bp)
*CALM1*	Fwd: AGGAAGCTTTCTCCCTGTTTG; Rv: TCCTCTTCACTGTCGGTGTCT	XM_024997842.1	214
*INHBA*	Fwd: TTGATATCGGAGAAGGTGGTG; Rv: CCCCCTCCTCTTCTTTCTTCT	XM_024990466.1	190
*INPPL1*	Fwd: GTGACCATACCCCATGACATC; Rv: GGACGTACTGACATGGCTGAT	NM_001191176.2	207
*NT5E*	Fwd: GGTCCAGTTAAAAGGCTCCAC; Rv: GTCTCCACCACTGACAAGGAA	NM_174129.4	250
*SCD*	Fwd: GTGGAGTCACCGAACCTACAA; Rv: GGAACCCTTTTCTTTGACAGC	NM_173959.4	229
*SDHB*	Fwd: AACTGTGGTCCTATGGTGCTG; Rv: CACATACATGTGTGGCAGAGG	NM_001040483.1	207
*RAB14*	Fwd: CAAGGAATCTCACCAATCCAA; Rv: AGCCTCAAGGAAAGCATCTTC	NM_001130754.1	179
*RPL19*	Fwd: GACCAATGAAATCGCCAATGC; Rv: ACCTATACCCATATGCCTGCC	NM_001040516	154

Abbreviations: AS, amplicon size (base pairs); Fwd, forward primer; Rv, reverse primer. * All primers were designed and validated by the authors. Each primer was used at a final concentration of 600 nM.

## Data Availability

All data pertinent to this work are contained in the article or available upon request. For all requests, please contact Kalidou Ndiaye (k.ndiaye@umontreal.ca).
